# Membraneless Organelles and Phase Separation in Tumours: Mechanisms and Prospects

**DOI:** 10.1111/cpr.70027

**Published:** 2025-04-11

**Authors:** Hao Yang, Zhong Chu, Shuwen Han, Yuefen Pan

**Affiliations:** ^1^ Department of Oncology, Huzhou Central Hospital Fifth School of Clinical Medicine of Zhejiang Chinese Medical University Huzhou Zhejiang Province China; ^2^ Department of Oncology, Affiliated Sir Run Run Shaw Hospital Zhejiang University School of Medicine Hangzhou Zhejiang Province China

**Keywords:** cancer, mechanism, Membraneless organelles, phase separation, therapy

## Abstract

Membraneless organelles (MLOs) are a type of subcellular compartment structure discovered in eukaryotes in recent years. They are mainly formed through the liquid–liquid phase separation (LLPS) and aggregation of macromolecular substances such as proteins or nucleic acids in cells. When cells are stimulated, they initiate a series of stress responses including gene transcription, RNA metabolism, translation, protein modification and signal transduction to maintain homeostasis. The dysregulation of these cellular processes is a key event in the occurrence and development of cancer. This article provides an overview of the structure and function of membraneless organelles, as well as the mechanisms of phase separation, to summarise the latest research progress on phase separation in tumours. It focuses on the role and molecular mechanism of LLPS in the development of tumours, with the aim of providing new theoretical references for developing drug action targets and innovative treatment strategies.

## Graphical abstract

The Salient and Novel Findings: This article summarises the latest research advancements in phase separation within tumours, with a particular focus on the role and molecular mechanisms of liquid–liquid phase separation (LLPS) in tumour progression. The aim is to provide new theoretical insights for the development of drug targets and innovative therapeutic strategies.

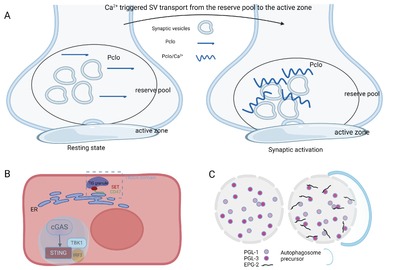

## 1 Introduction

Membraneless organelles (MLOs) are a type of subcellular compartment structure discovered in eukaryotic organisms in recent years. The liquid‐like assemblies within these organelles are formed through multivalent scaffold molecules via the process of liquid–liquid phase separation (LLPS). Scaffold molecules are mainly represented by intrinsically disordered proteins (IDPs) and, in some cases, by RNA. The low complexity and RNA‐binding domains of IDPs, along with the charged backbone and aromatic bases of RNA, facilitate multivalent interactions [1]. Due to the lack of cell membranes, these molecules can exchange components with their surrounding environment and respond to physiological stimuli rapidly and efficiently. This characteristic enables them to play a crucial role in physiological and pathological processes within cells. The MLOs that have been identified include the nucleolus, Cajal bodies, nuclear speckles, paraspeckles, promyelocytic leukaemia nuclear bodies (PML NBs), P‐bodies, stress granules (SGs), DNA damage body and histone locus body (HLB) [2] (Table 1).

**TABLE 1:** Membraneless organelles formed by phase separation.

Name	Location	Functions	References
Nucleolus	Nucleus	The site of ribosomal RNA (rRNA) production and ribosome subunit assembly.	[3]
Cajal bodies	Nucleus	Playing a crucial role in RNA metabolism and the formation of RNPs.	[4]
Nuclear speckles	Nucleus	The sites for splicing factor storage and modification.	[5]
Paraspeckles	Nucleus	Providing platforms for specific processes or functioning as molecular sponges by sequestering proteins and RNA.	[6]
PML	Nucleus	Functions as a suppressor of growth and transcription, it plays a crucial role in cell death, genomic stability, antiviral responses and cell division.	[7]
P‐bodies	Cytoplasm	Serving as critical sites for RNA degradation and storage, and considered to play a vital role in the regulation of translation.	[8]
Stress granules	Cytoplasm	Regulating translation, mRNA storage and stability, as well as cellular signalling in response to stress.	[9]

In this review, we describe the types of MLOs and recent insights into LLPS formation, regulation and function. We also emphasise the diverse impacts of biomolecular condensates (BMCs) on cell biology. Furthermore, we introduce how LLPS influences the occurrence and progression of different cancers. This information provides a theoretical foundation for understanding the significance of LLPS in tumour biology. We provide a detailed examination of MLOs that are closely associated with cancer patients, including PML NBs, autophagosomes and SGs. Finally, we evaluate the significant potential of LLPS in anticancer therapies and propose targeting phase separation as an innovative strategy for tumour treatment.

## 2 Phase Separation Functions in Cells

The concept of phase separation is a prevalent phenomenon in the field of physical chemistry. It describes the dynamic concentration of biomolecules from a homogeneous environment into a relatively dense phase, forming a sparse phase and a dense phase. In the field of biology, phase separation is a physicochemical phenomenon in which biological macromolecules, including cellular proteins and nucleic acids, undergo condensation to form liquid MLOs driven by weak multivalent interactions. Increasing evidence suggests that BMCs are membraneless assemblies that form dynamically and reversibly through LLPS, playing a vital role in numerous cellular functions [2].

### 2.1 Material Transportation

**FIGURE 1:**
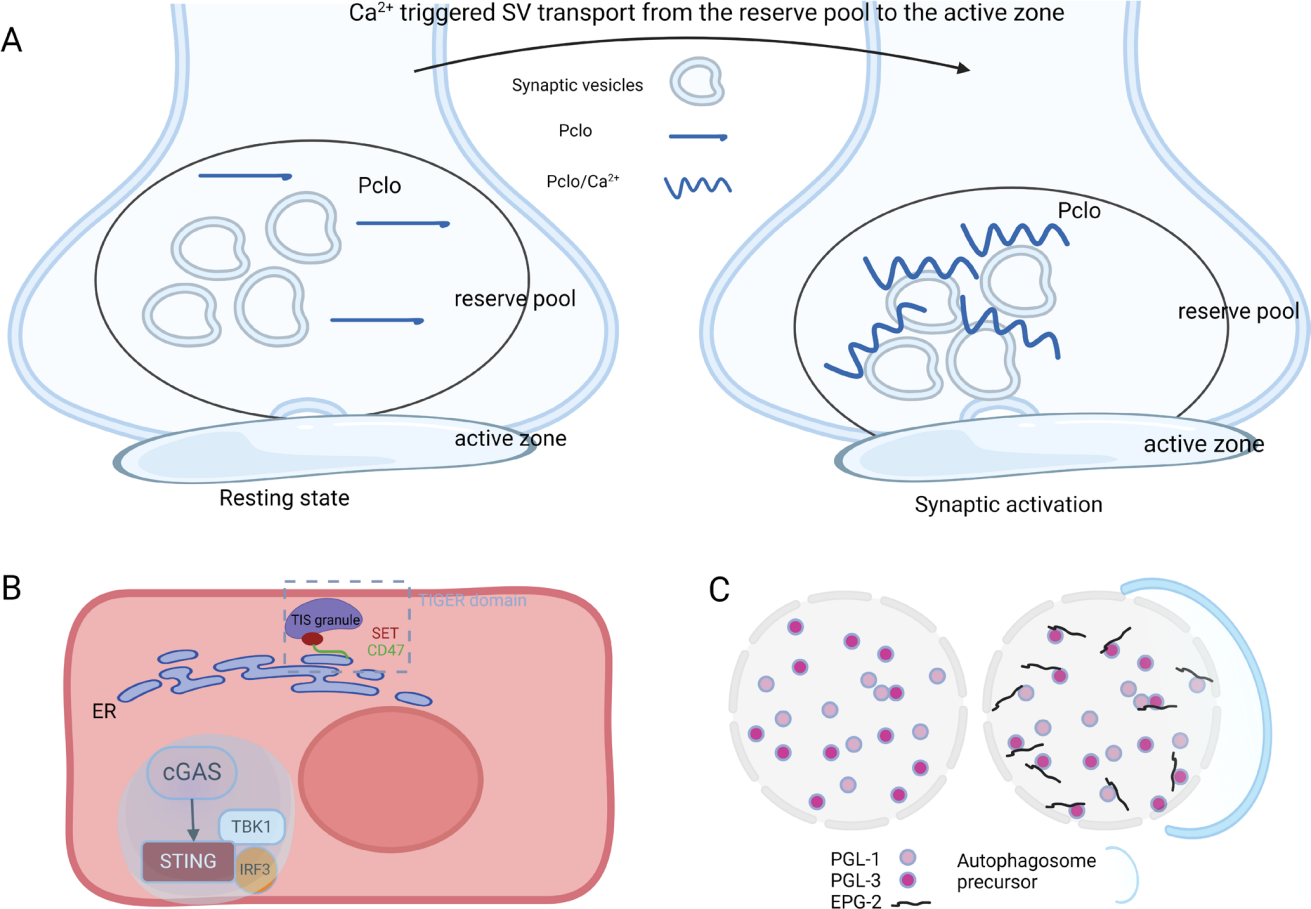
Phase separation in cell signal transduction. (A) Phase separation is involved in the process of SVs from an active state to a resting state; (B) Phase separation in cGAS‐STING‐TBK1 pathways and TIGER domain; (C) PGL‐1/3 and EPG‐2 play a crucial role in phase separation during the process of autophagy.

Transmembrane transport of substances is fundamental for cells to sustain normal life activities. Phase separation can participate in short‐distance transport within the premembrane during synaptic activation. Initially, synapsin undergoes phase separation to form reserve pool condensates, which recruit the calcium‐sensing dynamic scaffold protein Pclo. Subsequently, under the stimulation of calcium, Pclo's conformation changes and it phase separates from synaptic vesicles (SVs). This process enables Pclo to effectively extract SVs from the reserve area, thereby forming Pclo/vesicle transport condensates, which facilitate directional transport and adsorb vesicles from the reserve area to the surface of the active zone condensate. Phase separation is integral to organising all three types of condensates as well as regulating all SV pools [10] (Figure 1A).

Cubic membranes are present in nearly all cells of living organisms. These membranes often manifest under pathological conditions, such as infections, tumours and autoimmune diseases [11]. They are closely associated with cellular stressors, including abnormal protein synthesis and the production of reactive oxygen species. Xiaoyu Yu et al. [12] demonstrated that during the late stages of DNA virus infection, the intracellular cGAS‐STING pathway is activated. This activation results in the phase separation of the STING protein, leading to the formation of an endoplasmic reticulum cubic membrane structure that spatially ‘isolates’ STING‐TBK1 from the key transcription factor IRF3. Consequently, this process negatively regulates the cGAS‐STING pathway, inhibits innate immunity and ensures that innate immune responses do not become overactivated (Figure 1B).

Phase separation can regulate autophagy. Previous studies conducted in Zhang Hong's laboratory revealed that PGL‐1/3 aggregates into droplets after undergoing phase separation and is subsequently enveloped by the scaffold protein EPG‐2, forming a gelled state with low fluidity. This ultimately leads to the autophagosome enveloping the droplets, thereby inducing autophagy [13] (Figure 1C). Additionally, various RNPs resulting from phase separation can be anchored onto organelles via protein–protein interactions. These RNPs might also aggregate through phase separation and subsequently bind to the translocon complex on the membrane, thereby effectively facilitating protein transport [14].

### 2.2 Cell Adhesion

Cell junctions are specialised structural connections that form at the contact points of the plasma membrane between adjacent cells and between cells and the extracellular matrix. These junctions play a crucial role in cell diffusion and migration. Based on their structural and functional characteristics, they can be categorised into three types: tight junctions (TJ), anchoring junctions and communicating junctions. Junctional scaffold proteins generally include long intrinsically disordered regions (IDRs) and coiled‐coil domains, indicating that phase separation may be involved in the formation of these junctional complexes. However, in recent years, only focal adhesions (FAs) and TJs have provided experimental evidence supporting the occurrence of phase separation in adhesive connection assembly.

TJ consists of transmembrane proteins and cytoplasmic proteins. The ZO‐1 protein acts as a cytoplasmic protein, providing a scaffold effect. Dephosphorylation initiates its phase separation, transforming it into a condensed scaffold, which then facilitates the aggregation of TJ proteins to form a continuous TJ band. This process is essential for maintaining cell apical‐basal polarity and ensuring normal paracellular permeability in epithelial and endothelial tissues [15].

FAs represent adhesive connections between cells and the extracellular matrix, playing an important role in cell spreading and migration. Wang et al. [16] demonstrated that LIM domain‐containing protein 1 selectively enriches specific protein components via phase separation and promotes FAs maturation. Integrin adhesion complexes (IACs) are integrin‐based compartments associated with the plasma membrane, enabling cells to sense their surrounding environment. Phosphorylated focal adhesion kinase and focal adhesion kinase are integral components of IACs, and their phase separation is essential for initiating integrin aggregation, which facilitates the formation of new adhesions by IACs [17].

### 2.3 Signal Transduction

Recent studies underscore the significance of phase separation and condensate formation in the regulation of cell signal transduction. For instance, the T cell receptor (TCR) signalling pathway, which primarily operates in immune cells, undergoes spontaneous phase separation of downstream signalling proteins upon TCR activation. This process forms signal clusters that bolster specific output. Concurrently, certain kinases penetrate these clusters, intensifying the incoming signals. Given that TCRs are akin to receptor tyrosine kinases, which often mutate in cancer cells, the relevance to cancer is profound [18].

The centrosome functions as an essential membrane‐free organelle within the cell. It orchestrates DNA coordination to achieve partial replication through core regulatory factors such as Cyclin E‐Cdk2, DNA‐dependent protein kinase (DNA‐PK) and associated molecular complexes [19].

Additionally, signal transduction will be separated into its branches. For example, the RNA‐binding protein TIS11B forms TIS particles with the rough endoplasmic reticulum, creating the TIGER domain. This domain facilitates interactions between CD47, produced through the translation of CD47‐LU mRNA and the SET protein, thereby promoting the transport of CD47 to the cell membrane [20] (Figure 1B). SGS3 also forms condensates through phase separation both in vivo and in vitro. It interacts with RDR6 and RNA, driving the generation of siRNA within the cytoplasm. This process induces ribosome stalling, maintains epigenetic silencing of transposable elements and prevents cells from acquiring fatal mutations [21].

### 2.4 DNA Damage Response

DNA damage response (DDR) is essential for maintaining the integrity and stability of the genome. Abnormalities in DDR may lead to the emergence of oncogenic mutations. Among various types of DNA damage, DNA double‐strand breaks are the most lethal and they are repaired mainly through two pathways: homologous recombination (HR) and non‐homologous end joining (NHEJ). Current studies have shown that many molecules in the DDR pathway undergo phase separation. LLPS promotes the formation of repair compartments to facilitate HR and NHEJ in response to DNA damage [22]. For instance, within the nucleus, the MRE11‐RAD50‐NBS1 (MRN) complex is compartmentalised and concentrated within MRN‐interacting protein (MRNIP) condensates. These MRNIP condensates encapsulate the MRN complex, thereby enhancing its capability to efficiently capture and stabilise DNA breaks and promoting the activation of downstream molecules in the HR pathway [23]. Conversely, LLPS mediated by SUMOylated RING finger protein 168 (RNF168) limits RNF168 recruitment to DNA damage sites, reduces RNF168‐catalyzed H2A ubiquitination, inhibits 53BP1 in nuclear condensates and impairs the efficiency of the DNA repair NHEJ pathway [24]. LLPS can also affect DDR‐related pathways, such as Wnt/β‐catenin and Hippo pathways, altering the conformation or valence of related proteins, enhancing the properties of aggregates and thereby influencing tumour formation [25].

### 2.5 Maintaining Chromatin Organisation

Eukaryotes store their genetic information within the cell nucleus, where chromatin is organised and compacted through multi‐level structures. The three‐dimensional structure of chromatin is crucial for processes such as proper gene expression and DNA replication. Recent research indicates that phase separation is a critical factor in shaping higher‐order chromatin structures and facilitating their remodelling. At physiological salt concentrations, 10 nm nucleosomes can spontaneously condense chromatin into spheres via LLPS. H2A can form droplets with DNA in vitro, aiding in chromatin condensation. Furthermore, the linker histone H1 can phase separate from DNA in living cells through its C‐terminal IDR tail, and the inclusion of H1 can markedly enhance chromatin phase separation. In summary, LLPS directly drives the three‐dimensional organisation of chromatin at various levels, including compartments, topologically associated domains (TADs), loops and nucleosome condensation. Additionally, LLPS indirectly influences the three‐dimensional organisation of chromatin by modulating interactions between chromatin and key nucleosomes [26].

HLBs are BMCs that form at replication‐dependent (RD) histone genes within animal cells, playing a crucial role in regulating the transcription and processing of histone mRNAs. Hur et al. [27] studied the mechanism of growth and size of scaffold protein polymorphism in HLBs and found that HLBs can precisely control the size of nuclear bodies through phase separation. At the human chromosome 6 locus, three histone gene subclusters are interrupted by non‐histone genes along the length of the chromosome and are tightly connected to the fourth sequence in 3D space through LLPS, which participates in the assembly of nuclear bodies known as HLBs. Since HLBs exist throughout the cell cycle, changes in their composition regulate most of the cell cycle [28].

## 3 Phase Separation and Diseases

Recent studies have shown that abnormal phase separation of biomacromolecules is closely related to a variety of human diseases, including neurodegenerative disorders and cancer [29]. Most cell signalling proteins have IDRs, which are crucial for enabling LLPS. Table 2 offers an overview of potential targets and emerging strategies related to LLPS for the treatment of malignant tumours, providing a theoretical framework for the identification of effective target molecules.

**TABLE 2:** Transcription factors activated by LLPS and possible targets.

Tumour model	Complex	Cellular mechanisms (transcription factors)	References
Breast cancer	NUDT5	NUDT5 affects the phase separation process by regulating ATP synthesis in the nucleus of breast cancer cells.	[30]
	TAZ‐NANOG	TAZ and NANOG form phase‐separated droplets and enhance the transcription of multiple genes, including SOX2 and OCT4.	[31]
	β‐catenin 1	Phase separation in the cytoplasm inhibits Wnt signalling and promotes bone metastasis.	[32]
	Par3	Participating in JAM‐A phase separation to increase osmotic pressure and promote breast cancer cell migration	[33]
	HDAC6	Contributes to the formation of LLPS aggregates, activates transcription and inhibits TNBC cell proliferation.	[34]
	PML	The PML protein collaborates with HIF1A to induce the expression of metastatic genes and to promote the migration, invasion and metastasis of TNBC cells.	[35]
Gastric cancer	NAT10	Acetylation of SRSF2 by LLPS upregulates YTHDF1 expression and promotes gastric cancer cell proliferation and migration.	[36]
	SPOP	Promotes DAXX ubiquitination and induces cancer cell apoptosis.	[37]
	Matrilin‐3	Promotes phase separation and enhances the invasive ability of gastric cancer cells.	[38]
Hepatocellular carcinoma	Glycogen	Sequestration of Laforin‐Mst1/2 through phase separation inhibits Hippo pathway activation and promotes tumorigenesis.	[39]
	S6K1	Phosphorylation of eIF2α facilitates the formation of stress granules and supports the survival of liver cancer cells.	[40]
	URB1‐AS1	Promotes ferritin phase separation and alleviates sorafenib‐induced ferroptosis.	[41]
	ALDH3A1	Phase separation occurs, inducing resistance to ATO.	[42]
	YBX1	Phase separation can inhibit TPM4, restructure the tumour cell cytoskeleton and resist metastasis	[43]
	RNF214	Activates the C‐terminal ZnF/Ring‐dependent ligase activity of RNF214, thereby promoting the proliferation of HCC.	[44]
Lung cancer	P53	Activates downstream effectors involved in the DDR, which is involved in drug resistance.	[29]
	NONO	Promotes the recruitment and interaction among EGFR, TAZ and DNA‐PK, thereby enhancing the DNA repair process.	[45]
	USP42	Directing the integration of spliceosomal PLRG1 into nuclear speckles through phase separation promotes NSCLC progression.	[46]
	EML4‐ALK	Spatially restricting KRAS and amplifying KRAS oncogenic signalling.	[47]
	IGF2BP1	Enhances the stability of IGF2BP1, c‐Myc and E2F1. Facilitates the c‐Myc/MNX1‐AS1/IGF2BP1 complex to accelerate cell cycle progression and promote lung cancer cell proliferation.	[48]
Colorectal cancer	NOP533	Inhibits radiation‐induced p53 activation, thereby enhancing radioresistance.	[49]
	RNF168	Reduced RNF168‐catalysed H2A ubiquitination suppressed 53BP1 in nuclear condensates, ultimately decreasing DNA damage repair efficiency.	[24]
	EphA2	LLPS aggregates form on cell membranes, regulate ferroptosis and immune cell infiltration and contribute to colorectal cancer progression.	[50]
	PABPN	Contributes to the elongation of the 3′ UTR of CTNNBIP1 and inhibits cell proliferation and migration.	[51]
	DDX21	Regulates epithelial‐mesenchymal transition and promotes colorectal cancer cell metastasis.	[52]
	Fud‐RACK1	Inhibition of MTK1‐SAPK signalling pathway leads to reduced efficacy of 5‐Fu and drug resistance.	[53]
Pancreatic cancer	KMT2D	Enhance the stability of the WDR5 protein, promote the synthesis of the KMT2D‐enzyme complex and stimulate tumour growth.	[54]
	NUPR1	Promotes the formation of SGs and facilitates the growth of pancreatic cancer.	[55]
	MYC	Downregulating MYC transcription levels inhibits pancreatic cancer growth.	[56]
	SRPK2	The activation of the IGF1/PI3K/mTOR/S6K1 pathway mediates the formation of SGs and promotes the development of obesity‐related PDAC.	[57]
Immunity	cGAS	Recognition of DNA triggers the formation of liquid‐like condensates and activates the innate immune response.	[58]
	IFI16	As a viral DNA sensor, liquid IFI16 dot‐like structures are formed through the mechanism of LLPS.	[59]
	NLRP6	NLRP6 senses viral RNA and induces the formation of liquid NLRP6‐RNA condensates in vitro and in cells. Activation of the classic pathway of NLR inflammation.	[60]
	MAVS/STING	As RNA virus sensor, controlling downstream immunity.	[61]
	NF‐κB	Subunit p65 is rapidly sequestered, activating pro‐inflammatory cytokines.	[62]

### 3.1 Phase Separation in Breast Cancer

Breast cancer is the most prevalent and lethal malignant tumour among women worldwide. Oestrogen is closely associated with the occurrence and progression of breast cancer. ATP regulates oestrogen levels via phase separation, with NUDT5 being crucial for ATP production within the cell nucleus. Inhibiting NUDT5 diminishes ATP synthesis in the nuclei of BRCA cells, thereby reducing oestrogen activity and controlling the proliferation of BRCA cells [30]. Cancer stem cells (CSCs) are a rare subpopulation of tumour cells with the ability to self‐renew, asymmetrical cell division and differentiation. CSCs are believed to be the cause of metastasis, tumour recurrence, chemotherapy resistance and poor clinical outcomes [63]. In studies investigating chemotherapy resistance in breast cancer, researchers found that patients with increased matrix stiffness exhibited poor responses to neoadjuvant chemotherapy. In these cases, the PDZ‐binding motif (TAZ) and NANOG are more prone to forming phase separation droplets, which enhance NANOG's ability to transcribe multiple genes such as SOX2 and OCT4. This process increases the matrix stiffness of CSCs while simultaneously reducing chemotherapy sensitivity among breast cancer patients [31]. The dishevelled binding antagonist of beta‐catenin 1 acts as an inhibitor of β‐catenin 1 and is induced by transforming growth factor. It undergoes phase separation within the cytoplasm to form protein condensates that suppress Wnt signal transduction and facilitate bone metastasis [32].

Cell polarity protein Partitioning defective 3 (Par3) increases the osmotic pressure (OP) on exogenous chemokines by causing cytoskeletal depolymerisation and inducing JAM‐A phase separation, thereby promoting breast cancer cell migration [33]. Recently, Bing Lu and colleagues discovered that in triple‐negative breast cancer (TNBC), the levels of phosphorylated HDAC6 significantly increased in the nucleus. This increase contributed to the formation of LLPS aggregates, activating transcription and preventing further aggregation of phosphorylated HDAC6. Consequently, this mechanism could suppress TNBC cell proliferation, reduce tumour sphere formation capacity and enhance cellular apoptosis [34]. Additionally, the PML protein collaborates with HIF1A to enhance the expression of several metastasis‐driving genes, thereby facilitating the migration, invasion and metastasis of TNBC cells [35]. The inhibition of PML expression leads to reduced levels of MYC and PIM1 kinases, while promoting the accumulation of CDKN1B, which results in a stagnation of tumour cell growth and triggers cellular senescence [64].

### 3.2 Phase Separation in Gastric Cancer

Gastric cancer is the fifth most common cancer worldwide and the third leading cause of cancer‐related deaths. Recent studies have indicated that phase separation mechanisms contribute to its development and progression. NAT10, an acetyltransferase, undergoes LLPS to acetylate SRSF2. The acetylated SRSF2 subsequently binds to YTHDF1, enhancing its expression and thereby promoting the proliferation and migration of gastric cancer cells [36]. SPOP forms phase‐separated, membraneless clusters within nuclear speckles and has garnered extensive attention as a tumour suppressor gene. It promotes the ubiquitination of the Death domain‐associated protein (DAXX) via LLPS, effectively inducing apoptosis in cancer cells. When SPOP undergoes mutation, it loses the ability to form LLPS with DAXX, leading to the accumulation of a significant amount of DAXX protein. This process is associated with the early stages of gastric cancer [37]. Furthermore, research has shown that cancer‐associated fibroblasts can secrete matrilin‐3, which aids in phase separation, modifies the tumour microenvironment and enhances the invasive potential of gastric cancer cells [38].

### 3.3 Phase Separation in Hepatocellular Carcinoma

Hepatocellular carcinoma (HCC) is the sixth most common primary cancer in humans and is typically associated with a poor survival rate. Phase separation significantly contributes to the progression of HCC. Liu et al. [39] found that glycogen accumulation in the liver leads to phase separation, sequestering Laforin‐Mst1/2 within glycogen droplets. This inhibits Hippo activation and alleviates Yap inhibition, promoting tumorigenesis. In liver cancer cells, the activation of PI3K and the MAPK/p38 pathway leads to the activation of mTOR. This, in turn, phosphorylates eIF2α through S6K1, promoting the formation of SGs, which enables liver cancer cells to endure stress conditions [40]. Sorafenib is a targeted therapy that has gained widespread use in recent years for treating patients with advanced liver cancer. However, research indicates that liver cancer cells can mitigate sorafenib‐induced ferroptosis by promoting ferritin phase separation and decreasing cellular free iron levels through URB1‐AS1. This mechanism contributes to the resistance of liver cancer cells to sorafenib treatment [41]. The clinical response of HCC to arsenic trioxide (ATO) has traditionally been poor. Research indicates that the expression of PML is linked to prolonged survival and a lower recurrence rate following liver cancer resection. However, elevated PML expression can lead to resistance to ATO by upregulating ALDH3A1, which diminishes the anti‐tumour effect of ATO [42].

Intrahepatic metastasis poses a significant challenge in the treatment of HCC, necessitating extensive remodelling of the cytoskeleton. The actin cytoskeleton forms one of the primary networks within this system. As a regulatory protein, TPM4 promotes HCC metastasis by stabilising the actin cytoskeleton. Liu et al. [43] demonstrated that circASH2 promotes the LLPS of nuclear Y‐box binding protein 1, inhibits TPM4, remodels the tumour cytoskeleton and exhibits notable anti‐metastatic activity. Additionally, other studies have indicated that the phase separation mediated by the CC domain of RNF214 activates its C‐terminal ZnF/RING‐dependent ligase activity, thereby promoting proliferation, migration and metastasis in HCC. Conversely, the knockout of RNF214 can effectively inhibit these processes in HCC [44].

### 3.4 Phase Separation in Lung Cancer

Lung cancer is the leading cause of cancer death worldwide. DNA repair mechanisms are associated with tumorigenesis. Deficiencies in DNA damage repair can promote tumour development. Cancer cells may exploit specific DNA repair pathways to facilitate their own survival, ultimately contributing to drug resistance. P53 has been demonstrated to undergo LLPS following DNA damage, resulting in the formation of droplets that recruit and activate downstream effectors involved in the DDR [29]. However, mutations in p53 disrupt droplet formation, which subsequently promotes cancer development. Additionally, NONO's LLPS has been shown to facilitate the recruitment and enhance the interaction between the epidermal growth factor receptor (EGFR) and DNA‐PK, thereby promoting DNA repair processes and enabling tumour cells to develop radioresistance [46]. Furthermore, the recruitment of EGFR to the COX‐2 promoter increases COX‐2 expression, which can also contribute to tumour progression [45]. Additionally, NONO promotes LLPS of the transcriptional co‐activator with TAZ. TAZ exerts its transcriptional regulatory function through LLPS by forming nuclear condensates that drive gene expression programmes associated with cell proliferation, survival and invasion, ultimately contributing to non‐small cell lung cancer (NSCLC) resistance against chemotherapy and targeted therapies [65]. Moreover, USP42 is a deubiquitinase that guides the integration of spliceosome component proline‐rich coiled‐coil 1 (PLRG1) into nuclear speckles via phase separation, thus promoting the progression of NSCLC [66].

An important factor in NSCLC carcinogenesis is Anaplastic Lymphoma Kinase (ALK) fusion mutation, which is often associated with accelerated cancer progression [67]. Zhang et al. [47] demonstrated that the oncogenic fusion protein EML4‐ALK undergoes phase separation, spatially restricting KRAS and enhancing its signalling output, thereby amplifying the KRAS oncogenic signal. c‐Myc and E2F1 are critical players in numerous human cancers. Zhu et al. [48] revealed that the long noncoding RNA MNX1‐AS1 binds to IGF2BP1 and drives its phase separation, thereby increasing IGF2BP1's interaction with the 3′‐untranslated region of c‐Myc and E2F1 mRNAs to promote their stability. The positive feedback loop involving c‐Myc/MNX1‐AS1/IGF2BP1 accelerates cell cycle progression and fosters sustained proliferation of lung cancer cells.

### 3.5 Phase Separation in Colorectal Cancer

Colorectal cancer has emerged as the fourth most lethal cancer globally, following lung, gastric and breast cancers. LLPS plays a crucial role in the DDR, with enhanced DDR serving as a key molecular mechanism underlying tumour radioresistance. Research indicates that NOP533 undergoes LLPS within the nucleolus and inhibits radiation‐induced p53 activation, thereby enhancing radioresistance [49]. Ubiquitin E3 ligase RNF168 functions as an early‐response repair protein in the DNA double‐strand break repair pathway. RNF168 also exhibits LLPS, which restricts its recruitment to sites of DNA damage, diminishes RNF168‐catalyzed H2A ubiquitination, inhibits 53BP1 within nuclear condensates and ultimately compromises DNA damage repair efficiency. Sentrin/SUMO‐specific protease 1 acts as a deSUMOylating enzyme that reduces RNF168 SUMOylation and prevents its condensation in colorectal adenocarcinoma cells. This action enhances damage repair efficiency and increases the resistance of cancer cells to DNA‐damaging agents [24]. Recently, an increasing number of membrane‐associated molecules have been identified as functioning through phase separation mechanisms. EphA2 is aberrantly overexpressed in colorectal cancer and can form LLPS condensates on the cell membrane, which is positively correlated with the expression of ferroptosis‐related genes and immune cell infiltration, contributing to colorectal cancer progression [50]. The splicing factor SNRPD2 interacts with the glutamic acid‐proline domain of PABPN1, disrupting PABPN1's LLPS properties. This disruption weakens PABPN1's inhibitory effect on proximal poly(A) sites, leading to the shortening of the 3′ UTR of CTNNBIP1 and promoting cellular proliferation and migration [51]. Additionally, studies have shown that the RNA‐binding protein DDX21 undergoes phase separation within CRC cells, thereby regulating the epithelial‐mesenchymal transition processes and facilitating the metastasis of CRC cells [52]. 5‐Fluorouracil (5‐Fu) is one of the most commonly used adjuvant chemotherapy drugs in the treatment of colorectal cancer. Its metabolite, FUrd, is converted to FUTP and subsequently incorporated into RNA. This process promotes SGs assembly. RACK1 specifically localises to SGs and inhibits the MTK1‐SAPK signalling pathway and downstream apoptotic pathways in tumours; this inhibition leads to reduced efficacy of 5‐Fu and the development of drug resistance [53].

### 3.6 Phase Separation in Pancreatic Cancer

Histone lysine methyltransferase 2D (KMT2D) enhances the stability of the WDR5 protein via LLPS, facilitates the formation of the KMT2D‐enzyme complex, and catalyses the monomethylation of H3K4 to stimulate tumour growth [54]. In pancreatic cancer with the Kras G12D mutation, SGs formation can be promoted through NUPR1. ZZW‐115 inhibits the activity of NUPR1. Treatment with ZZW‐115 effectively reduces pancreatic tumour growth in Kras G12D mutant mice [55]. The LLPS inhibitor 1,6‐hexanediol can downregulate MYC transcription levels and inhibit pancreatic cancer growth, which may be related to the disruption of the MYC super enhancer's function. However, the specific mechanism requires further investigation [56]. SRPK2 mediates SGs formation in obesity‐related pancreatic ductal adenocarcinoma (PDAC) by activating the IGF1/PI3K/mTOR/S6K1 pathway. Silencing G3BP1 can inhibit SGs formation and reduce cellular resistance to oxidative stress. Inhibition of S6K1 selectively attenuates SGs and impairs the development of obesity‐related PDAC [57].

### 3.7 Phase Separation in Immunity

The immune system plays a pivotal role in preventing infectious diseases, autoimmune disorders and cancer. The formation of solid‐like higher‐order complexes has been considered a fundamental and ubiquitous mechanism underlying immune activation. However, with advancing research, the notion of liquid‐like higher‐order assemblies for eliciting immune responses has emerged. This perspective suggests that immune activation is not solely achieved through stable interactions but rather through dynamic binding processes that give rise to liquid‐like condensates. For instance, the recognition of DNA by cGAS leads to the formation of these liquid‐like condensates, thereby activating innate immune responses [58]. Nucleic acid immunity is crucial in combating pathogens and detecting cellular stress. Interferon‐induced protein 16 (IFI16) acts as a sensor for viral DNA. Upon recognising DNA, IFI16 undergoes LLPS to form punctate structures. Subsequent phosphorylation of the IFI16 IDR initiates a phase transition from the initial liquid droplets to final filamentous structures [59]. Beyond DNA sensing, phase separation also governs innate immune responses to RNA. Recently, LLPS has been identified as a novel mechanism that drives RNA virus‐induced NLRP6 inflammasome activation. NLRP6, which detects viral RNA, can trigger the formation of liquid‐like NLRP6‐RNA condensates both in vitro and in cells. This activation of the classic NLR inflammatory pathway is unexpected. The unexpected discovery of liquid‐like inflammasomes underscores an unforeseen role for LLPS in regulating RNA sensing, inflammation and antiviral immunity. Unlike solid structures, the liquid properties of inflammasomes allow them to integrate various stimuli and diversify their downstream outputs. For instance, cellular NLRP6 condensates can be activated by lipoteichoic acid (LTA), a molecular signature of Gram‐positive bacterial infection, or by double‐stranded RNA (dsRNA), a molecular pattern associated with certain RNA viruses, demonstrating the versatility of liquid droplets in detecting different signalling cues. Moreover, liquid NLRP6 condensates can initiate inflammasome formation or enhance IFN responses by recruiting the inflammasome adaptor protein apoptosis‐associated speck‐like protein or by interacting with the RNA helicase DHX15 [60]. Furthermore, LLPS regulates downstream immunity by inducing the formation of numerous cellular condensates. Certain RNA sensors relay signals to an adaptor protein known as MAVS. Like STING, MAVS is capable of undergoing LLPS both in vitro and within cells. Crucially, liquid MAVS droplets create an environment that ensures IRF3 is efficiently recruited and released, thereby facilitating the swift activation of antiviral responses. There is direct evidence indicating that phase separation plays a role in the regulation of IRFs and their downstream effectors. Upon translocation to the nucleus, IRF3 engages in LLPS with the promoter of the IFNB1 gene, driving the expression of type I IFN and antiviral genes [68]. In cells infected with respiratory syncytial virus (RSV), the NF‐κB subunit p65 is rapidly sequestered into perinuclear cytoplasmic puncta, which are synonymous with inclusion bodies. The trapped p65 is unable to translocate into the nucleus to activate downstream transcription of proinflammatory cytokine genes and other antiviral genes [62]. Two upstream regulators of the NF‐κB pathway, MAVS and MDA5, are recruited to RSV‐induced inclusion bodies as a mechanism to inhibit interferon‐β expression [61]. Although the field is still in its infancy, research into phase separation in immunology promises to offer new insights into understanding immune responses.

## 4 Prospects of Applying LLPS in Cancer Treatment

The potential of LLPS in cancer treatment has been extensively investigated by researchers. Ralaniten/EPI‐002 is the first drug that directly binds to the IDRs of the androgen receptor (AR) and has been tested in clinical trials [69]. Studies have also found that the small molecule compound ET0516 can effectively inhibit the phase separation formation of both wild‐type AR and drug‐resistant mutant AR [70]. BRD4 fosters malignant phenotypes across various cancer settings. Kondo et al. [71] designed and synthesised a cyclopropenone compound, ACP‐1n, which was found to inhibit BRD4 function by targeting its phase separation properties, leading to a significant reduction in colorectal cancer cell growth and nuclear size. Ming et al. [56] applied 1,6‐hexanediol to pancreatic cancer cells, eliminating the LLPS process, which significantly inhibited the proliferation of these cells and induced cell death. Chloroquine, by blocking autophagy through impairing autophagosome–lysosome fusion, suggests that the clinical use of chloroquine in combination with anticancer therapy could improve tumour cell sensitivity [72]. The newly developed SRC‐3 inhibitor, SI‐2, significantly inhibits breast cancer growth through direct interaction with SRC‐3. It also eliminates cancer stem‐like cells, thereby preventing lung tumour initiation. Additionally, SI‐2 can inhibit the formation of LLPS and reduce NSD2‐mediated H3K36me2 modification, which enhances myeloma cell resistance [73]. Nexturastat A, as a phosphorylated HDAC6 LLPS inhibitor, has been demonstrated to induce chromatin structure remodelling and transcriptional profile changes, effectively inhibiting TNBC tumour growth [34].

**FIGURE 2:**
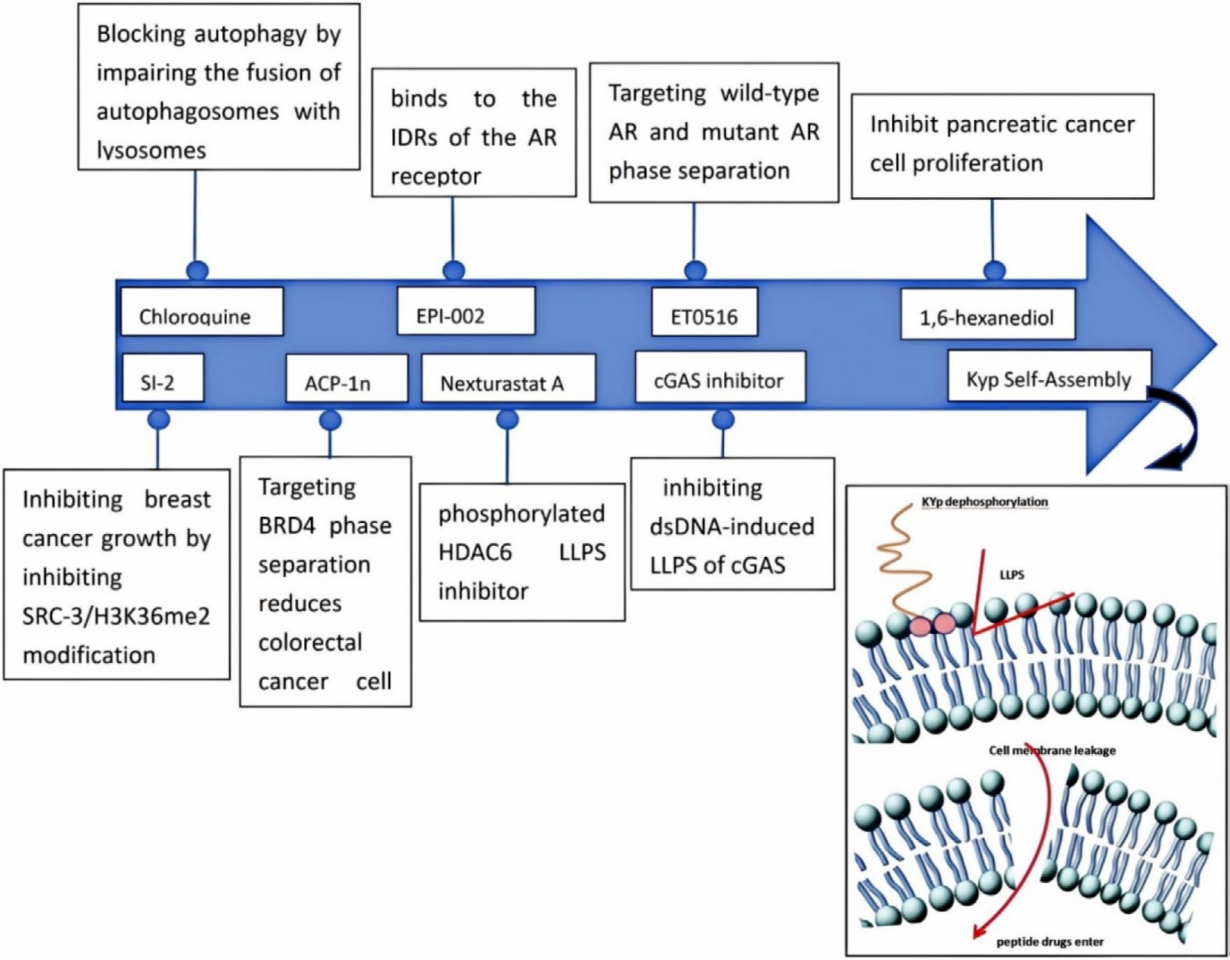
Small molecule inhibitors related to LLPS in prevalent clinical malignancies.

As we enter the immune era, immunity has become a topic of considerable interest. Xiaoquan Wang and colleagues identified a cGAS cyclic peptide inhibitor that specifically targets the protein‐DNA interface between cGAS and double‐stranded DNA (dsDNA). This inhibitor disrupts their interaction, thereby inhibiting dsDNA‐induced LLPS of cGAS. Such findings may provide new strategies for managing immune‐related side effects associated with immunotherapy [74]. Shuangquan Gou et al. [75] utilised gelatin to induce the liquid–liquid microphase separation of silk fibroin protein, thereby preparing a series of micro‐nanoparticle (GSC) drug delivery systems with varying particle sizes and surface charges. These systems efficiently encapsulate and facilitate intracellular delivery of small molecule chemical drugs, large molecule protein drugs and nucleic acid drugs. They demonstrated a significantly better therapeutic effect than free drugs in an in vivo breast cancer lung metastasis model. Peptides are of great interest in cancer therapy. Ruochen Guo et al. [76] have developed an in vivo self‐assembly strategy by inducing the LLPS of lipids and proteins on cell membranes through the self‐assembly of Kyp, which enhances the intracellular uptake and anticancer effects of peptide drugs (Figure 2).

## 5 Summary

Currently, there remains much to explore regarding the dynamic processes involved in MLOs. It is essential to investigate how to integrate the physicochemical properties of LLPS with clinical treatments and identify key molecules that regulate MLOs in order to effectively combat cancer. A major challenge is the lack of understanding of the dynamic characteristics of LLPS and the complexity of its associated signalling pathways. Since LLPS involves IDPs or IDRs that lack stable isolated structures, this structural plasticity allows IDPs/IDRs to exist in a variety of conformations. Developing effective and specific LLPS inhibitors that can selectively target cancer cells is another challenge. In summary, phase separation is a nascent and rapidly evolving field. We believe that as research progresses, we can better understand the biological functions of phase separation and be able to provide more effective treatments for cancer.

## Author Contributions

**Hao Yang:** writing – original draft (lead), software (lead), writing – review and editing (equal). **Zhong Chu:** writing – review and editing (equal). **Shuwen Han:** conceptualisation (supporting), writing – review and editing (equal). **Yuefen Pan:** formal analysis (lead), writing – review and editing (equal).

## Ethics Statement

The authors have nothing to report.

## Consent

The authors have nothing to report.

## Conflicts of Interest

The authors declare no conflicts of interest.

## Data Availability

The authors have nothing to report.
